# Identification and validation of ferroptosis-related gene SLC2A1 as a novel prognostic biomarker in AKI

**DOI:** 10.18632/aging.205669

**Published:** 2024-03-20

**Authors:** Huaying Wang, Yuanyuan Li, Xinran Liu, Yonggui Wu

**Affiliations:** 1 Department of Nephrology, The First Affiliated Hospital of Anhui Medical University, Hefei, Anhui 230022, PR China; 2 Center for Scientific Research of Anhui Medical University, Hefei, Anhui 230022, PR China

**Keywords:** acute kidney injury, ferroptosis, SLC2A1, bioinformatics analysis, prognosis

## Abstract

Background: Emerging evidence reveals the key role of ferroptosis in the pathophysiological process of acute kidney injury (AKI). Our study aimed to investigate the potential ferroptosis-related gene in AKI through bioinformatics and experimental validation.

Methods: The AKI single-cell sequencing dataset was retrieved from the GEO database and ferroptosis-related genes were extracted from the GENECARD website. The potential differentially expressed ferroptosis-related genes of AKI were selected. Functional enrichment analysis was performed. Machine learning algorithms were used to identify key ferroptosis-related genes associated with AKI. A multi-factor Cox regression analysis was used to construct a risk score model. The accuracy of the risk score model was validated using receiver operating characteristic (ROC) curve analysis. We extensively explored the immune landscape of AKI using CIBERSORT tool. Finally, expressions of ferroptosis DEGs were validated *in vivo* and *in vitro* by Western blot, ICH and transfection experiments.

Results: Three hub genes (BAP1, MDM4, SLC2A1) were identified and validated by constructing drug regulatory network and subsequent screening using experimentally determined interactions. The risk mode showed the low-risk group had significantly better prognosis compared to high-risk group. The risk score was independently associated with overall survival. The ROC curve analysis showed that the prognosis model had good predictive ability. Additionally, CIBERSORT immune infiltration analysis suggest that the hub gene may influence cell recruitment and infiltration in AKI. Validation experiments revealed that SLC2A1 functions by regulating ferroptosis.

Conclusions: In summary, our study not only identifies SLC2A1 as diagnostic biomarker for AKI, but also sheds light on the role of it in AKI progression, providing novel insights for the clinical diagnosis and treatment of AKI.

## INTRODUCTION

AKI, characterized by a sudden and rapid decline in renal function, is a condition that is known to have high mortality rates. It has been observed that approximately 10–15% of hospitalized patients and over 50% of patients in the intensive care unit develop AKI [1, 2]. The International Association of Nephrology reports that AKI affects around 13 million individuals worldwide annually, leading to approximately 1.7 million deaths each year [3]. It is important to note that AKI survivors face a significantly increased risk of developing chronic kidney and end-stage renal diseases. Despite the increasing understanding of AKI and the improvement of clinical support and adjuvant treatment measures, the prognosis of AKI remains poor and the mortality rate remains high. Early detection and intervention in the treatment of AKI, along with early control of the condition, can prevent irreversible kidney damage. Currently, the diagnosis of AKI primarily relies on blood creatinine and urine output. However, these traditional indicators do not effectively reflect early decline in renal function and are easily influenced by other factors. Although there are existing AKI-related biomarkers such as neutrophil gelatinase-associated lipocalin and cystatin C that can indicate changes in renal function during the early stages of AKI, their limited availability and detection methods have hindered widespread application in clinical practice. Therefore, it is urgent to identify novel diagnostic biomarkers and find personalized treatment strategies for AKI.

Ferroptosis is an identified form of iron-dependent programmed cell death, distinct from apoptosis, necroptosis. It involves lipid peroxidation, reactive oxygen species production, and mitochondrial dysfunction. The kidney is especially vulnerable to redox imbalance. Multiple bodies of evidence indicate the potential involvement of ferroptosis in the pathophysiological mechanisms underlying AKI via diverse pathways [4]. For example, in folic acid induced AKI mice models, apoptosis inhibitor could not reduce the tubular epithelial cells injury, while Fer-1 could effectively reduce renal tubular cell death [5]. In IR-induced AKI mice models, ALR has been proven to prevent the damaged kidney by modulating Xc/GSH/GPX4 signaling and scavenging ROS to inhibit ferroptosis [6, 7]. In AKI mice models, ACSL4 expression was upregulated in the renal tissues, and mechanistically, HIF-1α directly bound to the promotor of the ACSL4 gene and regulated ACSL4 transcription. Knockout of ACSL4 mitigated renal damage by decreasing inflammatory response and suppressing immune cell infiltration and ferroptosis [8]. However, ferroptosis-related genes of AKI remain to be further explored.

To identify novel AKI diagnostic biomarkers, we used bioinformatics and machine learning methods to screen ferroptosis-related genes associated with AKI and verified through *in vitro* and *in vivo* experiments. This study will not only identify diagnostic biomarker for AKI, but also sheds light on the role of it in AKI progression, providing novel insights for the clinical diagnosis and treatment of AKI.

## RESULTS

### Cell clustering analysis based on scRNA-seq sample

**Figure 1:**
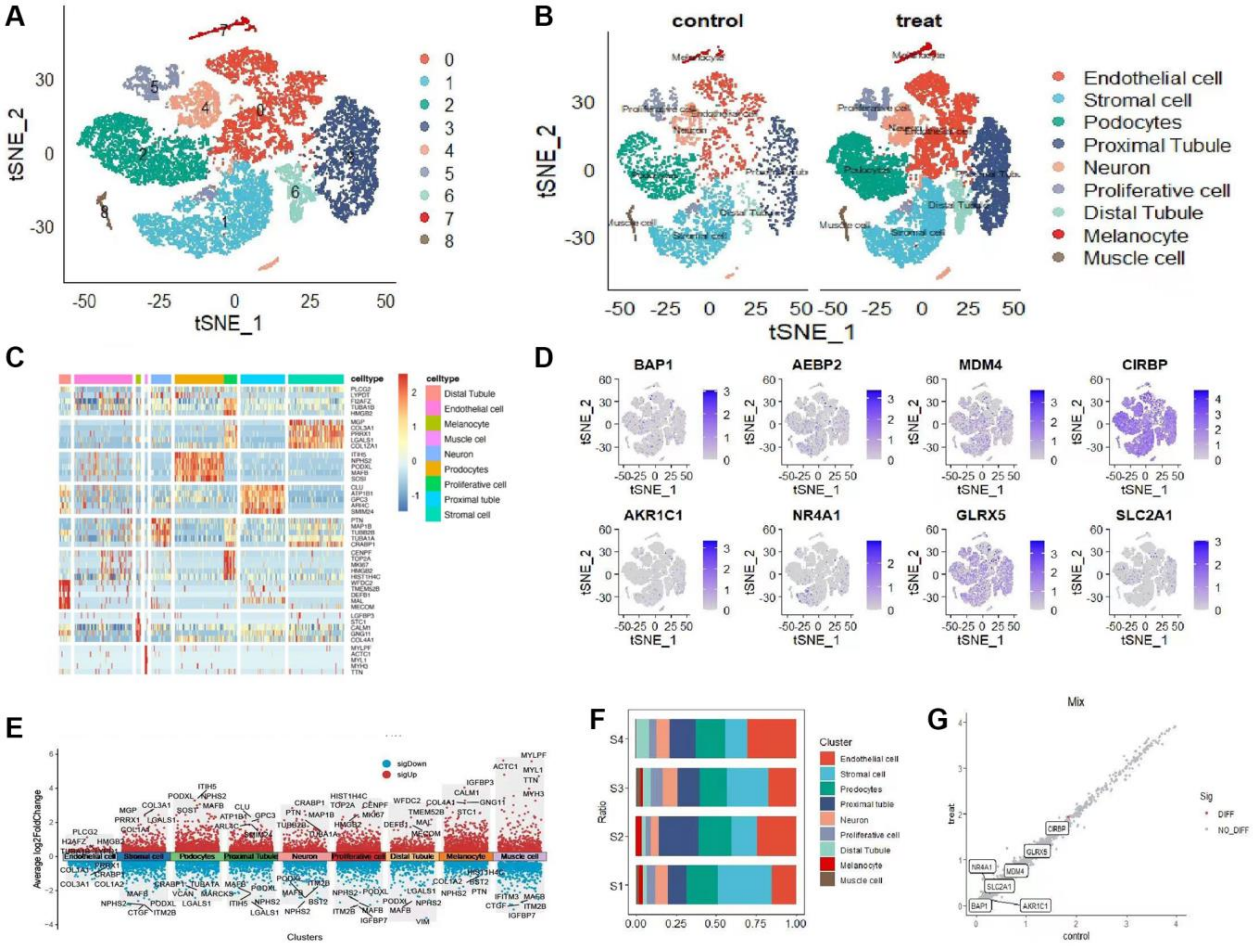
**Single-cell clusters were identified based on scRNA data from AKI patients.** (**A**) The tSNE diagram shows 9 clusters and the expression of iron-related marker genes associated with iron-induced cell death. (**B**) The tSNE diagram illustrates the distribution of 8 cell subsets after clustering. (**C**) The heat map shows the expression levels of the top 5 marker genes in each subpopulation. (**D**) The tSNE map displays the distribution of 8 marker genes. (**E**) The volcano map demonstrates the expression patterns of the first 5 marker genes within each subpopulation. (**F**) Subgroup proportions and cell counts are depicted in 4 sample groups. (**G**) The distribution of gene expression is compared between the two groups.

After the initial screening, logarithmic normalization and dimensionality reduction methods were employed on the scRNA-seq dataset. By utilizing the t-SNE approach, a total of 9 distinct cell subtypes were discovered (Figure 1A). The distribution of each cell subtype is visualized in the t-SNE plot shown in (Figure 1B). The 9 cell subtypes primarily comprise of Endothelial cells, Stromal cells, Podocytes, Proximal Tubules, Neurons, Proliferative cells, Distal Tubules, Melanocytes, and Muscle cells. In order to identify genes that are differentially expressed among these clusters, we employed the R package “FindVariableFeatures.” This methodology enabled the identification of genes that display significant variations across the distinct cell types. The heatmap in (Figure 1C) displays the most prominently expressed differentially regulated genes in each cellular cluster, with the stromal cell cluster showing notably elevated expression levels of COL3A1, MGP, PRRX1, LGALS1, and COL1A1 (Figure 1D) displays the 8 marker genes utilized for single cell clustering analysis. Furthermore, volcano plots were employed to illustrate the top 5 highly expressed and lowly expressed genes in each of the nine cell subtypes (Figure 1E). (Figure 1F) illustrates the proportional distribution of cell subtypes in the four examined samples, showcasing the prevailing presence of Endothelial cells, Podocytes, and Stromal cells within the tissue. Furthermore, (Figure 1G) demonstrates the distribution of gene expression levels between the control and disease groups. Genes exhibiting a linear pattern on the diagonal line indicate consistent expression patterns in both groups.

### Analysis of genetic differences related to ferroptosis

**Figure 2:**
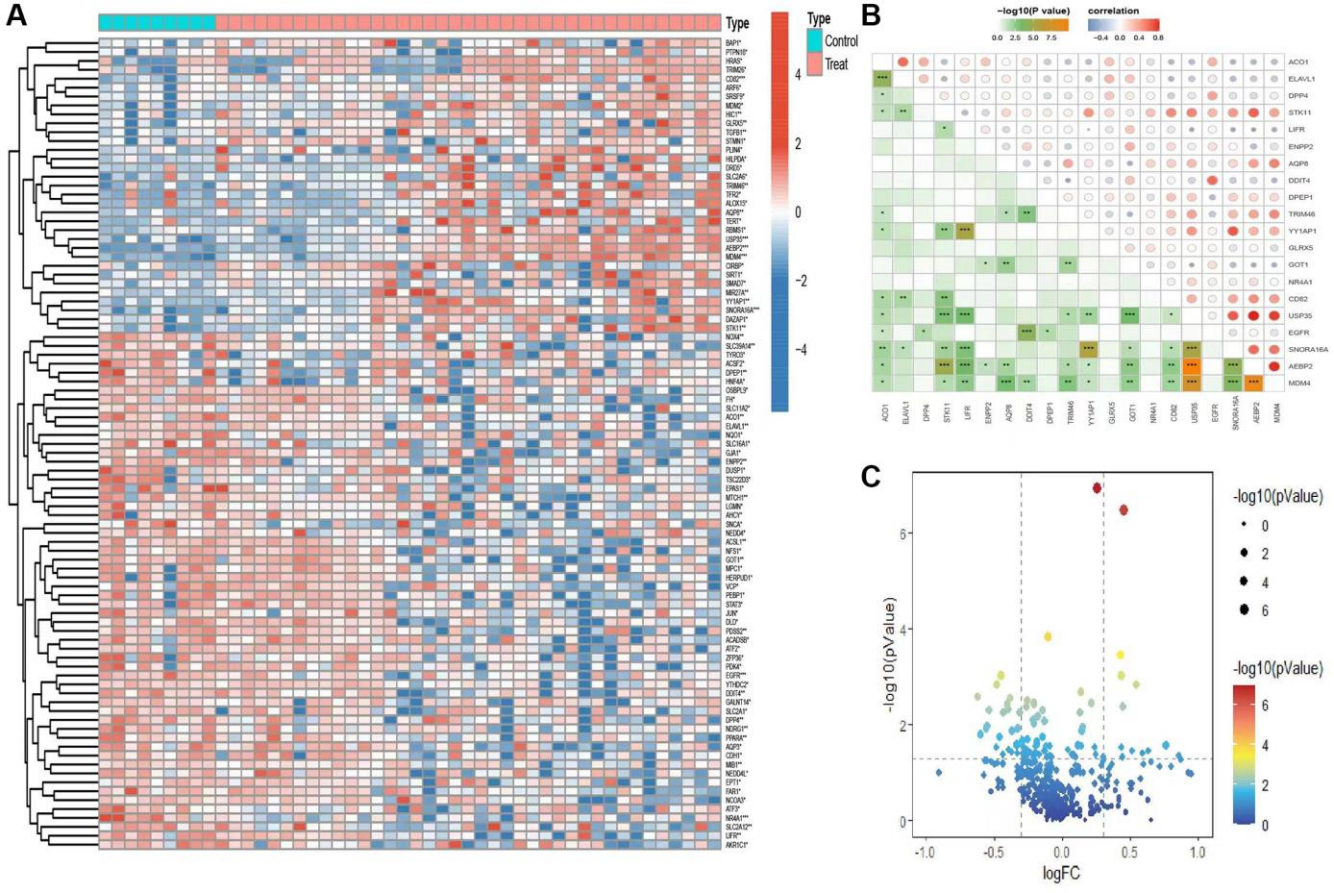
**Analysis of genetic variations associated with iron-related mortality.** (**A**) Heatmap shows distribution of gene differences related to iron-related mortality between the control group and the disease group. (**B**) Analysis of differential gene correlations. (**C**) The volcano map depiction of differential gene expression in ferroptosis.

We acquired the GEO dataset and extracted ferroptosis-associated genes from the GENECAED website to conduct an investigation. Specifically, our analysis focused on assessing the differential expression of genes implicated in iron-dependent cell death among patients diagnosed with AKI, when contrasting the cohorts of healthy individuals and those afflicted with the disease. The heat map presented in (Figure 2A) depicts the differential expression pattern of genes related to ferroptosis. It visually represents the distribution of these genes and their associated functions. (Figure 2B) illustrates the correlation between the top 20 genes exhibiting differential expression. This analysis highlights a positive correlation between the gene USP35 and AEBP2, while a negative correlation is observed between LIFR and YY1AP1. Additionally, the volcano plot depicted in (Figure 2C) presents supplementary evidence indicating the varied expression of genes implicated in ferroptosis.

### Construction of predictive models

**Figure 3:**
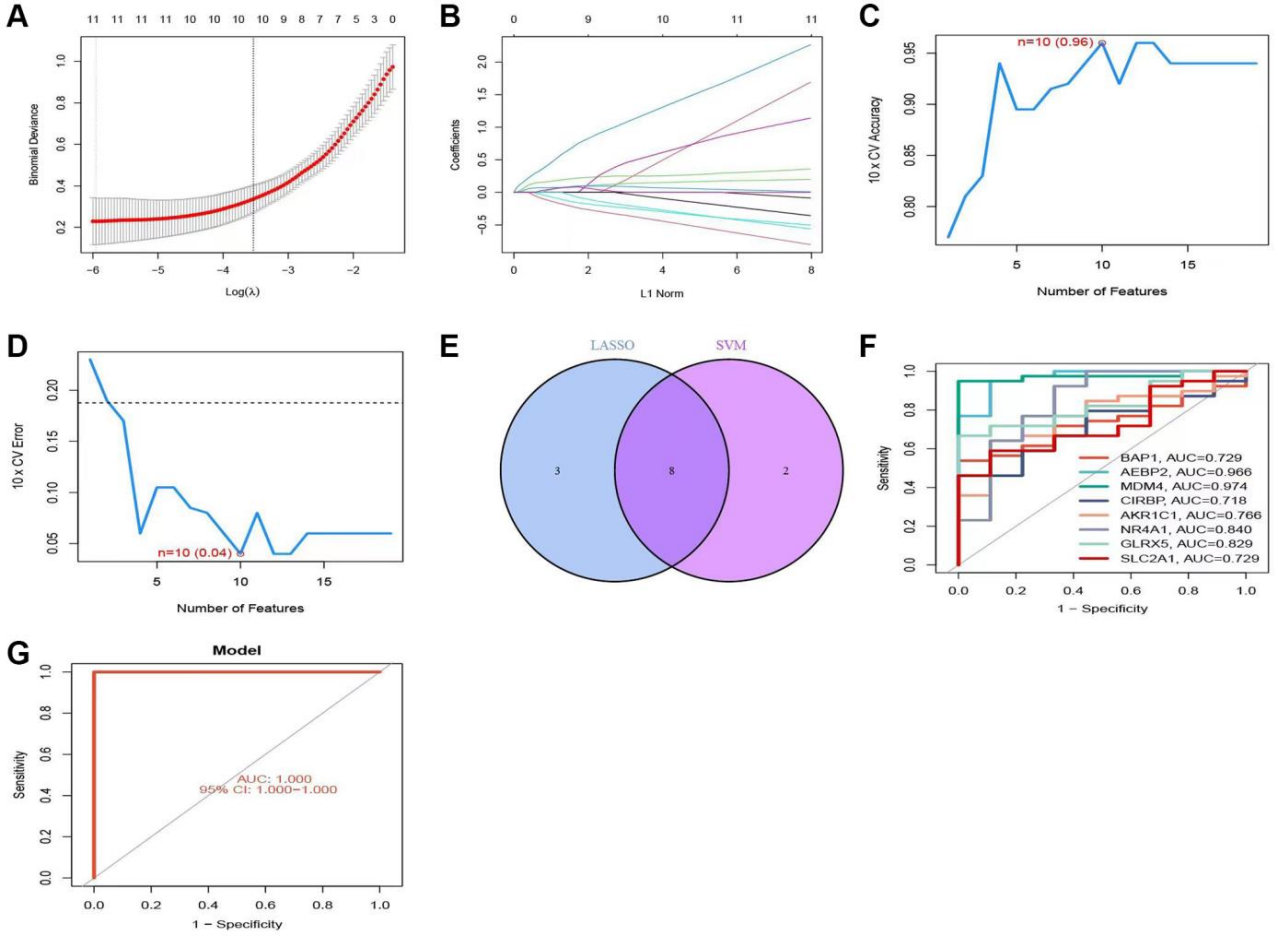
**Construction of the prediction model.** (**A**, **B**) Trajectories and distributions of each independent variable for lambda. (**C**, **D**) Reverse cumulative distribution of absolute residual for the SVM model. (**E**) Intersection of two model genes. (**F**) ROC curve showing the prediction results of the model genes. (**G**) Accuracy of the SVM model demonstrated by the ROC curves.

The GSE98320 dataset was employed to develop a predictive model. Lasso Cox regression analysis was utilized to identify a selection of 11 genes associated with iron-induced mortality. These genes were then used for the construction of the prediction model, as shown in (Figure 3A, 3B). The support vector machine (SVM) algorithm was subsequently employed for the development of a prediction model for AKI, utilizing a set of 10 genes associated with iron-induced mortality (Figure 3C, 3D). In order to ensure the precision of the genes utilized for model development, the intersection of genes derived from both models was determined, resulting in the identification of 8 shared genes (Figure 3E). The predictive diagnostic results of the eight target genes for AKI in the validation set are displayed in (Figure 3F). The area under the curve (AUC) value was computed for each individual gene and for the comprehensive diagnosis. The AUC values for the individual genes were as follows: BAP1: AUC = 0.729; AEBP2: AUC = 0.966; MDM4: AUC = 0.974; CIRBP: AUC = 0.718; AKR1C1: AUC = 0.766; NR4A1: AUC = 0.840; GLRX5: AUC = 0.829; SLC2A1: AUC = 0.729. The ROC curve analysis indicated that the AUC value for the comprehensive diagnosis utilizing the eight target genes was 1, with a 95% confidence interval of 1–1 (Figure 3G).

### Pathway enrichment analysis

**Figure 4:**
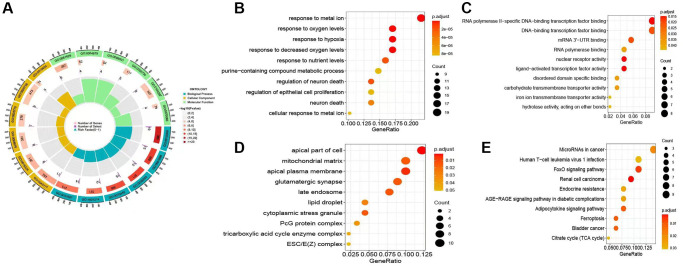
**Analysis of the differentially expressed genes (DEGs) related to AKI.** (**A**) Circular plot illustrating the Gene Ontology (GO) analysis, highlighting the potential gene functions of the DEGs associated with ferroptosis and its influence on the occurrence and progression of AKI. (**B**–**D**) Bubble plot representing the GO analysis in terms of biological processes (BP), cellular components (CC), and molecular functions (MF). (**E**) Kyoto Encyclopedia of Genes and Genomes (KEGG) analysis of the DEGs associated with ferroptosis and AKI.

The DEGs related to ferroptosis were determined using an adjusted *p*-value less than 0.01 and a |logFC| greater than or equal to 1.5, between the normal and AKI groups. In this manner, duplication rates are reduced. The possible involvement of these DEGs AKI was investigated using GO and KEGG enrichment analyses. The GO enrichment analysis demonstrated a significant enrichment of immune-related biological processes among the DEGs identified (Figure 4A). The KEGG enrichment analysis further revealed a notable enrichment of various pathways associated with cell proliferation and the cell cycle in the DEGs (Figure 4B–4E).

### Immune analysis

**Figure 5:**
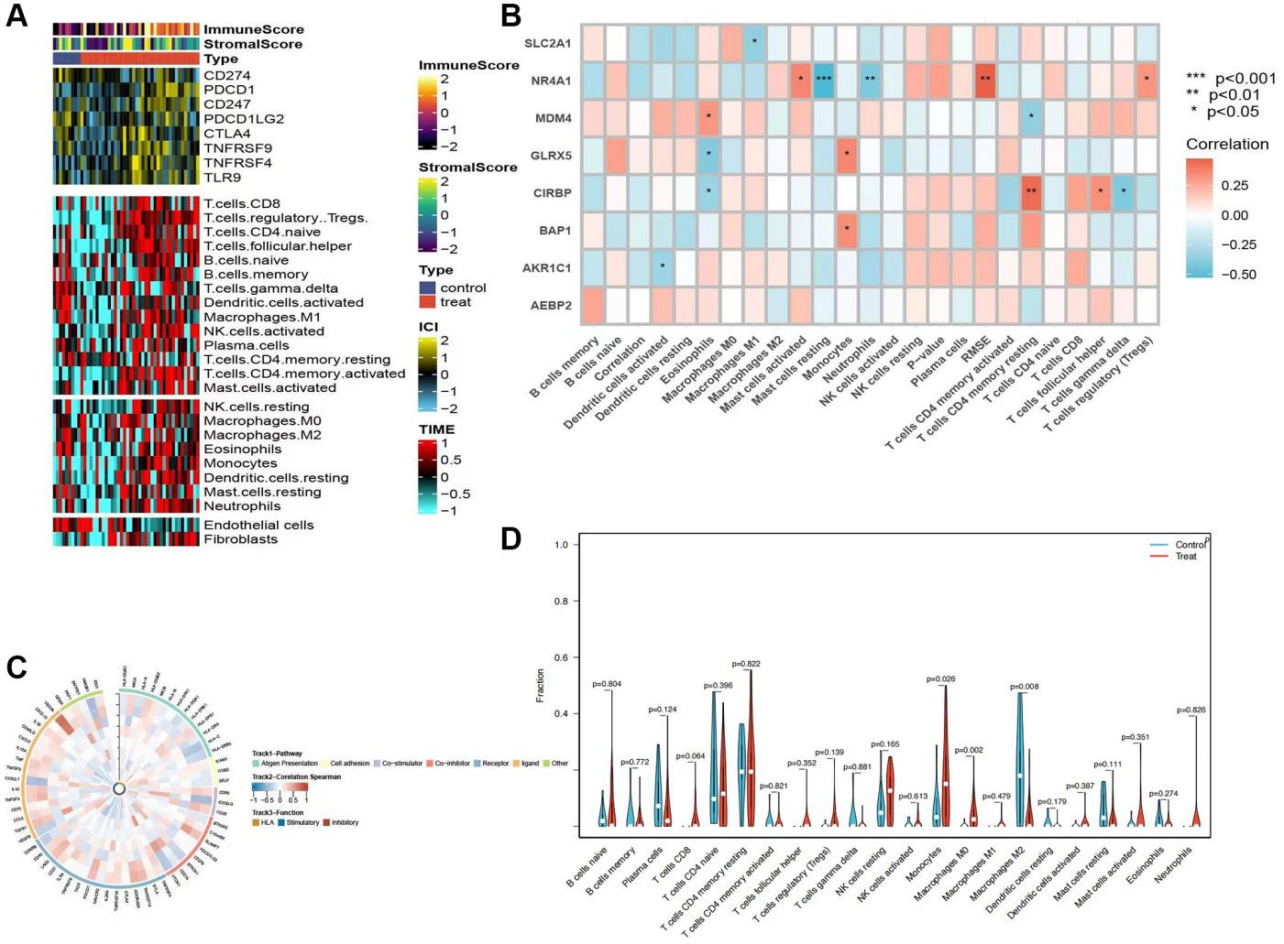
**Analysis of immune infiltrations.** (**A**) Heatmap showing the results of immune cell infiltration in the tumor microenvironment (TME) in AKI using multiple algorithms, including data from the TIMER and MCP-counter platforms. TME-related scores are displayed in the top bar. (**B**) Correlations between eight hub genes and 22 immune-related cells. (**C**) Correlation analysis between nine hub genes and 75 immune-associated genes. (**D**) Comparison of the proportions of 22 immune-related cells between control and treated groups.

After examining the distribution of immune and stromal cell infiltration within both normal and diseased cohorts, a notable disparity was observed, with the disease group demonstrating a greater prevalence of immune and stromal cell infiltration compared to the normal group (Figure 5A). Moreover, the study revealed a greater prevalence of CD8 T cells, Tregs cells, CD4 naïve cells, and memory B cells within the disease group. Furthermore, an investigation was conducted to assess the correlation between model genes and immune cell populations. In (Figure 5B, 5C), a strong correlation was observed between several genes, including NR4A1, CIRBP, MDM4, GLRX5, and immune cells. Specifically, gene NR4A1 exhibited positive correlations with Mast cells resting, RMSE, and Tregs, but negative correlations with Mast cells resting and Neutrophils. Additionally, the comparison of 22 immune-related cells between the two groups revealed that the normal group had a higher abundance of Macrophages M2 cells, while Monocytes and Macrophages M0 cells were more prevalent in the disease group (Figure 5D). These findings suggest significant relationships between specific genes and immune cell populations, highlighting potential implications in disease mechanisms [9]. M0 macrophages, characterized as unactivated and immature, perform the crucial function of phagocytosing and clearing cellular debris, initiating inflammatory responses [10]. Conversely, M2 macrophages are primarily anti-inflammatory and involved in the reparative and regenerative processes [11], generating anti-inflammatory mediators like IL-10 and TGF-β, thus alleviating inflammation and promoting tissue repair and regeneration [12]. In summary, M2 macrophages have distinctive roles in immune responses and inflammation, while M0 macrophages remain in an undifferentiated state with neutral characteristics [13]. Manipulating the ratio of M2 macrophages offers potential for regulating immune and inflammatory responses, providing a foundation for potential treatment strategies for AKI [14].

### Gene set enrichment analysis (GSEA) of model genes

**Figure 6:**
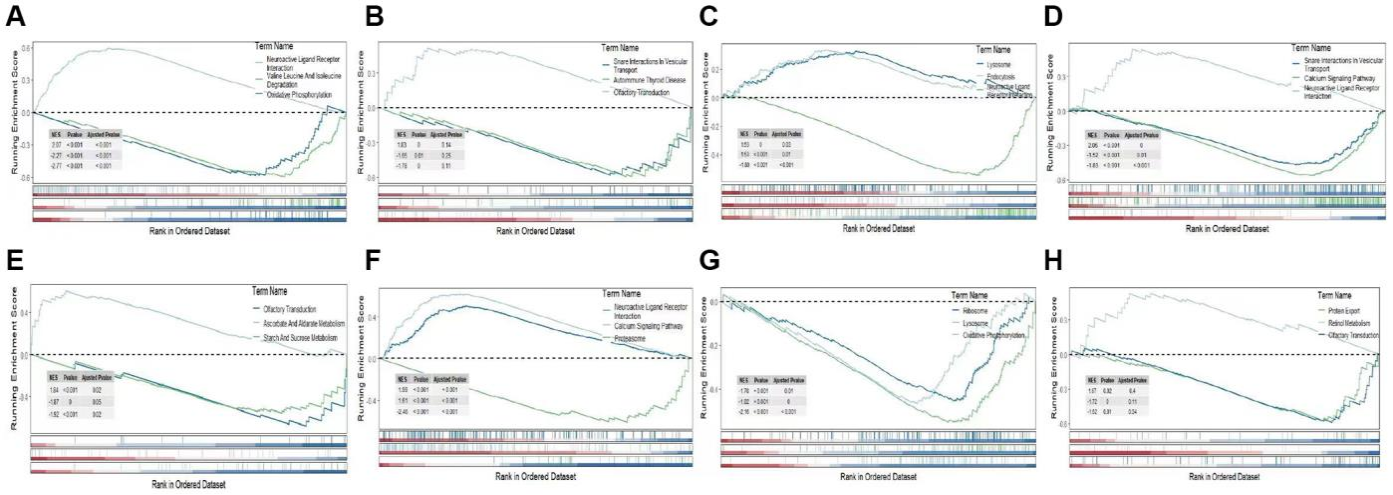
**Pathway analysis of model genes.** (**A**) AEBP2 enrichment pathway. (**B**) AKR1C1 enrichment pathway. (**C**) BAP1 enrichment pathway. (**D**) CIRBP enrichment pathway. (**E**) GLRX5 enrichment pathway. (**F**) MDM4 enrichment pathway. (**G**) NR4A1 enrichment pathway. (**H**) SLC2A1 enrichment pathway. The upper panel shows the enrichment pathway analysis for genes with high expression, while the lower panel represents the enrichment pathway analysis for genes with low expression.

**Figure 7:**
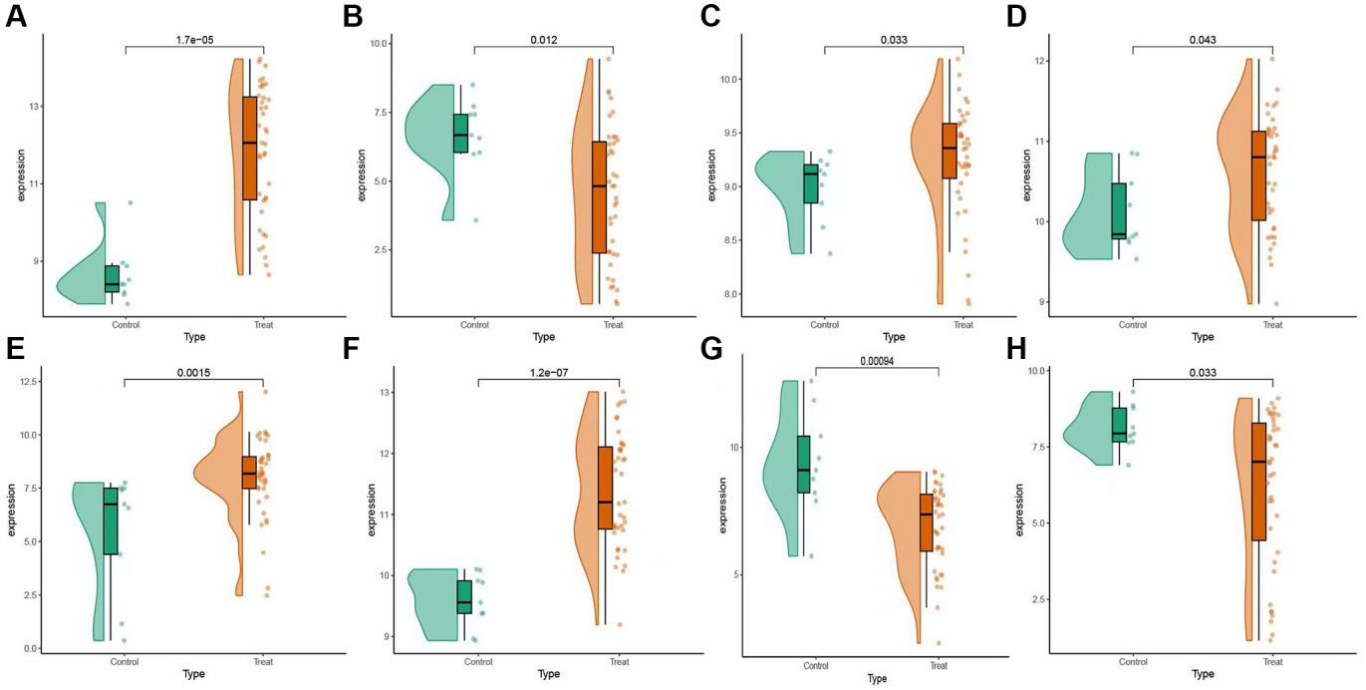
**Expression levels of selected candidate genes.** (**A**) AEBP2 gene expression level. (**B**) AKR1C1 gene expression level. (**C**) BAP1 gene expression level. (**D**) CIRBP gene expression level. (**E**) GLRX5 gene expression level. (**F**) MDM4 gene expression level. (**G**) NR4A1 gene expression level. (**H**) SLC2A1 gene expression level.

Gene Set Enrichment Analysis (GSEA) was conducted to investigate variances in gene function among the KEGG pathways of the selected target genes. Notably, AEBP2 displayed significant associations with the following pathways: neuroactive ligand receptor interaction, valine leucine isoleucine degradation, and oxidative phosphorylation (Figure 6A). The involvement of AKR1C1 was demonstrated in vesicular transport snare interactions, autoimmune thyroid disease, and olfactory transduction pathways (Figure 6B). In (Figure 6C), BAP1 appeared to be significantly enriched in the lysosome, endocytosis, and neuroactive ligand signaling pathways. In (Figure 6D), CIRBP exhibited connections with vesicular transport involving SNARE interactions, the calcium signaling pathway, and neuroactive ligand receptor interactions. GLRX5 displayed enrichment in pathways related to olfactory transduction, ascorbate and aldarate metabolism, and starch and sucrose metabolism (Figure 6E). MDM4 was found to be associated with pathways such as neuroactive ligand receptor interaction, calcium signaling and proteasome pathways (Figure 6F). NR4A1 exhibited enrichment in pathways such as ribosome, lysosome, and oxidative phosphorylation (Figure 6G). SLC2A1 was identified to be enriched in pathways related to protein export, retinol metabolism, and olfactory transduction (Figure 6H). An examination was carried out to determine the levels of expression of these 8 selected genes in both the disease group and the normal group (Figure 7A–7H). The findings indicated that SLC2A1 (Figure 7A), AEBP2 (Figure 7C), BAP1 (Figure 7D), CIRBP (Figure 7E), and GLRX5 (Figure 7F) exhibited significantly higher expression levels in the disease group. Conversely, MDM4 (Figure 7B), AKR1C1 (Figure 7G), and NR4A1 (Figure 7H) demonstrated elevated expression levels in the normal group.

### Construction and pharmacological regulation analysis of ceRNA networks

**Figure 8:**
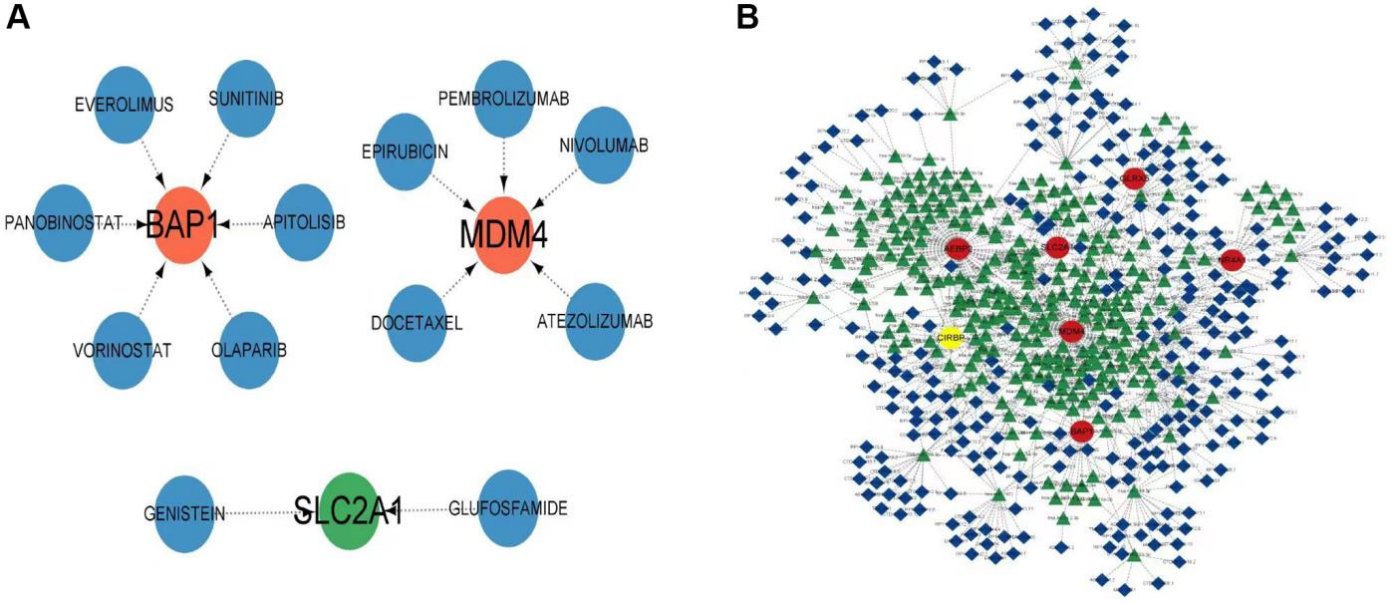
**Construction of competing endogenous RNA (ceRNA) network and regulation by drugs.** (**A**) Drug regulatory network of model genes. (**B**) The ceRNA network associated with model genes. Up-regulated entities are indicated in red, while down-regulated entities are indicated in yellow.

Cytoscape was utilized for the construction of the CeRNA network and analysis of drug regulation. This investigation unveiled connections between the model genes and various drugs, such as everolimus, sunitinib, panobinostat, apitolisib, vorinostat, and olaparib, particularly in relation to BAP1. Additionally, the association of MDM4 with epirubicin, pembrolizumab, nivolumab, atezolizumab, and docetaxel was observed, whereas SLC2A1 showed an association with genistein and glufosfamide (Figure 8A). Furthermore, the interactions among model genes, miRNA, and LncRNA were further investigated, with an elevated expression indicated by the color red and a diminished expression represented by the color yellow (Figure 8B).

### SLC2A1 and ACSL4 were overexpressed in AKI and promote HK-2 cell apoptosis

**Figure 9:**
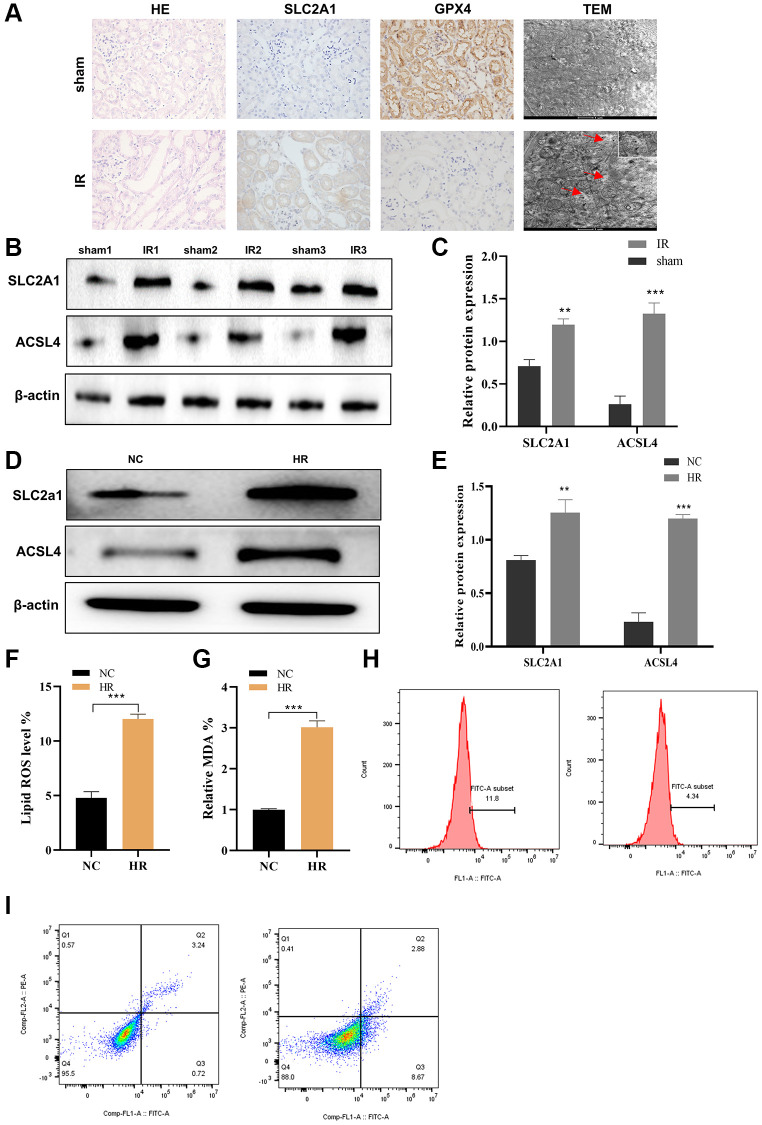
**SLC2A1 and ACSL4 were overexpressed in AKI and promote HK-2 cell apoptosis.** (**A**) HE and immunohistochemistry of SLC2A1, GPX4 and TEM in renal ischemia-reperfusion injury model; (**B**) The protein level of SLC2A1 and ACSL4 in renal ischemia-reperfusion injury model and normal control model was verified by Western blot; (**C**) Relative quantification of SLC2A1 and ASCL4 in B; (**D**) The protein level of ACSL4 and SLC2A1 in HR induced AKI model and was verified by Western blot; (**E**) Relative quantification of these proteins in D; (**F**) The lipid ROS level was verified in HR induced AKI model and normal control model; (**G**) The MDA level was verified in HR induced AKI model and normal control model; (**H**, **I**) The apoptosis rate was verified in HR induced AKI model and normal control model. ^*^p < 0.05, ^**^p < 0.01, ^***^p < 0.001. Abbreviations: MDA: Malondialdehyde; ROS: Reactive Oxygen Species.

**Figure 10:**
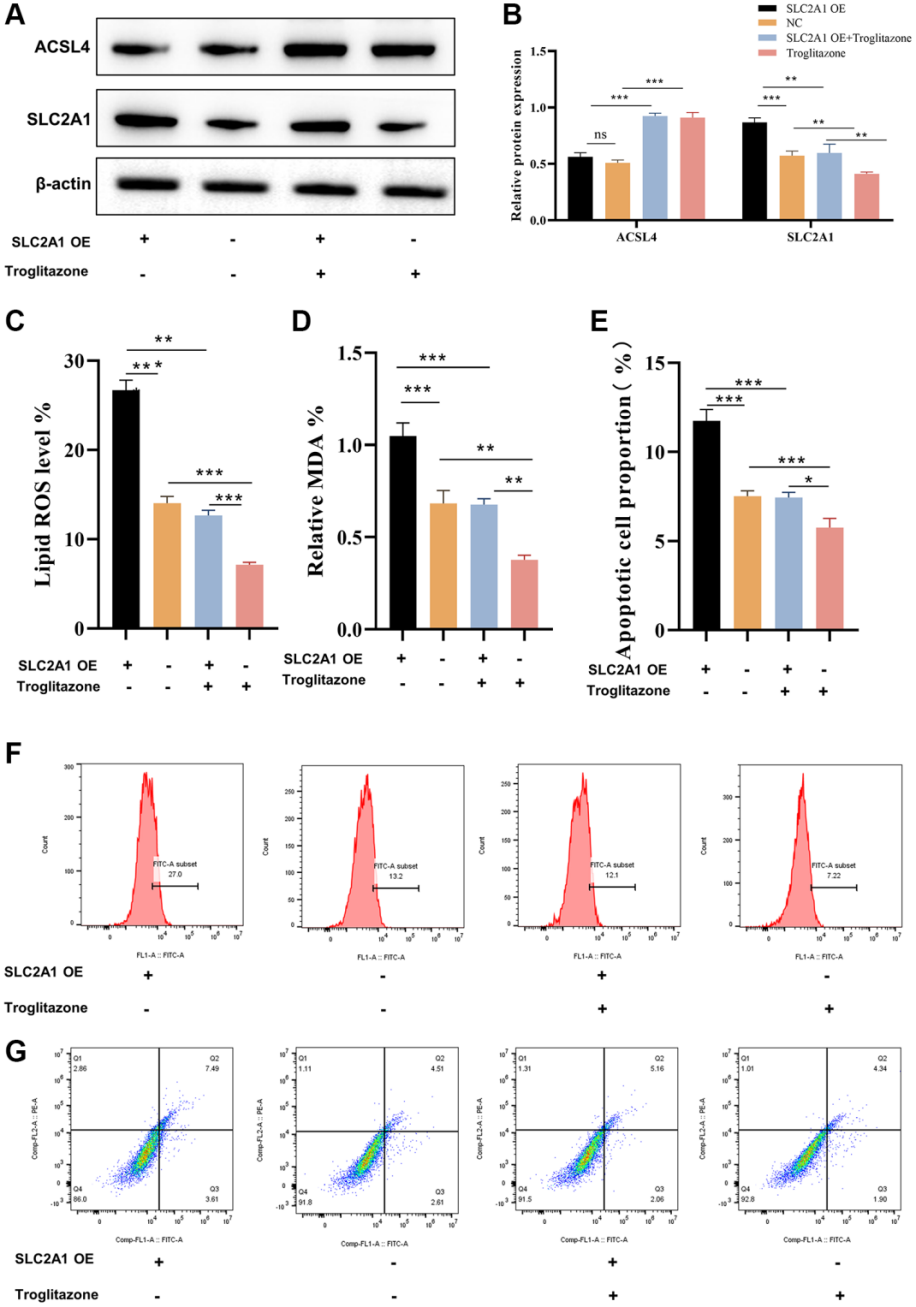
**SLC2A1 promotes HK-2 cell apoptosis and ferroptosis via ACSL4.** (**A**, **B**) The protein level of ACSL4 and SLC2A1 in HR induced AKI mode when overexpressed SLC2A1, overexpressed SLC2A1+T, or T was verified by Western blot and relative quantification of these proteins; (**C**) The lipid ROS level was verified in HR induced AKI mode when overexpressed SLC2A1, overexpressed SLC2A1+T, or T; (**D**) The MDA level was verified in HR induced AKI model when overexpressed SLC2A1, overexpressed SLC2A1+T, or T; (**E**–**G**) The apoptosis rate was verified in HR induced AKI model when overexpressed SLC2A1, overexpressed SLC2A1+T, or T. ^*^p < 0.05, ^**^p < 0.01, ^***^p < 0.001. Abbreviations: T: Troglitazone; MDA: Malondialdehyde; ROS: Reactive Oxygen Species.

To study the involvement of SLC2A1 in AKI, a mouse model of ischemia-reperfusion injury was developed using C57BL/6 mice. Following euthanasia, immunohistochemistry and Western blot analyses were conducted on the isolated kidney tissues to examine the expression of SLC2A1. It was observed that the ischemia-reperfusion kidney injury model led to renal damage and caused ferroptosis, which were identified by HE staining and TEM, and also a marked elevation in the levels of SLC2A1 and a decrease in the level of GPX4 expression in the renal tissues of mice (Figure 9A). The Western blot results of SLC2A1 and ACSL4 were consistent with immunohistochemistry mentioned above (Figure 9B, 9C). Furthermore, verification of the hypoxia-reoxygenation model was conducted employing the HK-2 cell line. The results revealed that hypoxia-reoxygenation induced an elevation in the expression of ACSL4, and SLC2A1 in the HK-2 cells (Figure 9D, 9E). Moreover, elevated lipid peroxidation and accumulation of malondialdehyde (Figure 9F, 9G) were detected, along with an augmented rate of cellular apoptosis (Figure 9H, 9I). Our experimental findings confirmed the crucial role of ACSL4 in ferroptosis and revealed the involvement of ferroptosis in the hypoxia-reoxygenation model. To further understand how SLC2A1 can regulate cell fate through ACSL4, we employed a specific ACSL4 inhibitor called Troglitazone. Our results demonstrated that overexpression of SLC2A1 in the hypoxia-reoxygenation model led to the upregulation of ACSL4, and SLC2A1 expression in the HK2 cells (Figure 10A, 10B). Accompanied by heightened lipid peroxidation and malondialdehyde accumulation in HK-2 cells (Figure 10C, 10D), an increased rate of cell apoptosis was observed (Figure 10E–10G). Additionally, inhibition of ACSL4 was observed to prevent the alterations in reactive oxygen species (ROS), malondialdehyde (MDA), and cellular apoptosis induced by SCL2A1. Taken together, our results propose that SLC2A1 drives the demise of renal proximal tubular epithelial cells via ACSL4-mediated ferroptosis in the hypoxia-reoxygenation model, ultimately contributing to the impairment of renal functional cells in cases of AKI.

## DISCUSSION

Acute kidney injury (AKI) is a growing global health issue characterized by increasing incidence rates [15]. This condition not only compromises patients’ physical health and survival but also places a significant strain on healthcare systems [16]. The efficient management of AKI heavily relies on the timely diagnosis and intervention, which play a vital role in ensuring effective interventions [17]. The timely identification of AKI allows for immediate implementation of therapeutic interventions, resulting in a more favorable prognosis [18]. Currently, close attention is given to monitoring urine output, blood chemistry markers, and renal function in order to facilitate early detection and prevent further damage to the kidneys [19]. Nevertheless, the achieved outcomes have been somewhat restricted to date [20]. The clinical importance of AKI has been acknowledged by the medical community of late, prompting endeavors to refine our comprehension, early identification, and efficient treatment approaches for this ailment [21]. Research on diagnostic and prognostic biomarkers in the field of medicine has gained significant traction [18]. Recent studies have highlighted the critical role of ferroptosis in AKI development and progression. Many ferroptosis-related genes have been found to be closely related to AKI.

SLC2A1 (solute carrier family 2 member 1), also referred to as glucose transporter type 1 (GLUT1), is a significant gene within the human genome that holds a pivotal position in glucose metabolism [22]. Its primary function revolves around facilitating the transportation of glucose across cellular membranes, thereby guaranteeing a sufficient supply of glucose as an energy source for various physiological processes [23]. Meanwhile, SLC2A1 is also a programmed cell death-related gene that plays important roles in ferroptosis [24]. Previous studies have investigated its role as a prognostic and immunotherapeutic marker in lung adenocarcinoma [25], where it was found to be aberrantly expressed in numerous cancers. Furthermore, SLC2A1 has shown prognostic significance and plays a role as an immune marker in various other cancers [26–28]. In the study of non-tumor diseases, evidence suggests that high expression of SLC2A1 is associated with type 2 diabetes and its complications [29], as well as chronic inflammation [30]. The interaction of SLC2A1 may influence the onset of chronic kidney disease by regulating ferroptosis [31]. However, the diagnostic and prognostic significance of SLC2A1, as a gene associated with ferroptosis in AKI has not yet been fully understood.

In this study, we conducted an extensive bioinformatics analysis of the SLC2A1 gene, which is known to be associated with the process of ferroptosis. This gene’s involvement in the development and progression of AKI was also explored. Moreover, we delved into its potential correlation with various clinical characteristics, immune infiltration, and immune checkpoints.

Our study focused on exploring the connection between ferroptosis and AKI. By utilizing the Human Protein Atlas, we identified four genes that were specifically expressed in AKI tissue and involved in the process of ferroptosis. Through analysis of single-cell RNA-sequencing data, we also identified several genes that were associated with ferroptosis. Among these genes, we selected eight (BAP1, AEBP2, MDM4, CIRBP, AKR1C1, MR4A1, GLRX5, SLC2A1) to develop a new risk signature using differential analysis, univariate Cox regression, lasso regression, and multivariate Cox regression methods.

To assess the predictive value of the risk signature, we employed the GEO AKI dataset. Based on the calculated risk scores, patients were classified into high- and low-risk groups using the median value as the cutoff. Our findings demonstrated that the low-risk group had significantly better prognosis compared to the high-risk group. Additionally, the risk score was independently associated with overall survival, as observed in both univariate and multivariate Cox regression models.

Tubular cell death plays a crucial role in the early stages of AKI and triggers inflammation through the release of chemokines and damage-associated molecular patterns from dying cells [32]. Various forms of cell death contribute to tubular cell loss in AKI, including ferroptosis, which is characterized by the accumulation of iron-dependent lipid hydroperoxides [33]. Ferroptosis has been shown to be significant in AKI mouse models induced by ischemia-reperfusion injury or oxalate crystal, contributing to renal tubule necrosis [34].

While apoptosis, pyroptosis, and necroptosis are well-established forms of cell death regulated by the immune system [35–37], it remains unclear if ferroptosis has a similar physiological role, whether through sensitization or induction via intrinsic or extrinsic mechanisms [38]. Given the complexities of ferroptosis and its involvement in various metabolic pathways, it is plausible that the immune system targets specific key elements of the ferroptosis process. The SLC2A1 study aims to analyze immune changes in AKI during immunotherapy to identify biomarkers that can accurately predict the immune response and guide treatment strategies [39]. The ultimate goal is to enable clinicians to select the most effective therapy and identify suitable recipients who will benefit from it, thereby avoiding treatment delays and achieving improved outcomes in AKI management.

We extensively explored the immune landscape of AKI based on risk features associated with specific risk genes (SRGs). Our findings indicate that the high-risk subgroup of patients exhibits increased immune cell infiltration. However, we observed that monocytes, RMSE, and resting CD4T cells are the predominant immune infiltrating cell types in the high-risk population. These cells have been shown to induce immune evasion in cancer immunotherapy, which poses a disadvantageous response to immune treatment. Therefore, we propose that immunotherapy may be more effective for patients in the low-risk group. Supporting our viewpoint, our analysis of the IMvigor210 and GSE78220 cohorts aligns with previous studies, as low-risk patients appear to derive greater benefits from immunotherapy compared to high-risk patients.

Based on our experimental results, we observed a significant upregulation of SLC2A1 and ACSL4 expression in the kidneys of mice subjected to an ischemia-reperfusion injury model of AKI. Additionally, in the HK-2 cell line, we found that hypoxia-reoxygenation resulted in upregulation of ACSL4, and SLC2A1 expression. These changes were accompanied by lipid peroxidation and an increase in cell apoptosis rate.

The significant upregulation of SLC2A1 and ACSL4 expression in the AKI model suggests their involvement in the pathogenesis of AKI. SLC2A1 is responsible for glucose uptake, and its overexpression in AKI may contribute to metabolic dysregulation [40]. ACSL4, on the other hand, plays a role in lipid metabolism and has been implicated in ferroptosis, a type of regulated cell death characterized by iron-dependent accumulation of lipid peroxides. Our findings suggest that ACSL4-mediated ferroptosis may be involved in the death of renal proximal tubular epithelial cells, ultimately leading to the disruption of renal functional cells in AKI.

Furthermore, our experiments using a specific inhibitor of ACSL4 demonstrated that blocking ACSL4 can inhibit the changes in reactive oxygen species (ROS), malondialdehyde (MDA) accumulation, and cell apoptosis induced by SLC2A1 overexpression. This suggests that SLC2A1 promotes the death of renal proximal tubular epithelial cells through ACSL4-mediated ferroptosis in the hypoxia-reoxygenation model [41].

Overall, our findings provide insight into the role of SLC2A1 and ACSL4 in the pathogenesis of AKI. They highlight the involvement of ACSL4-mediated ferroptosis in the death of renal cells and the disruption of renal function, However SLC2A1 needs to be further studied in future studies using knockout animals. Further research on the molecular mechanisms underlying SLC2A1 and ACSL4 dysregulation in AKI may uncover potential therapeutic targets for the treatment of this condition.

## CONCLUSIONS

Elevated SLC2A1 gene expression demonstrates a robust correlation with prognosis and heightened invasive capacity in iron-induced cellular apoptosis, indicating its potential contribution to the progression of AKI through modulation of immune cell infiltration. This investigation highlights SLC2A1 as a potentially valuable diagnostic and prognostic biomarker in AKI patients, offering novel functional implications and presenting it as a promising target for both diagnostic and therapeutic interventions.

## MATERIALS AND METHODS

### Transcriptome data acquisition and processing

The AKI single-cell RNA sequencing (scRNA-seq) dataset was obtained from the Gene Expression Omnibus (GEO) database with the accession number GSE149687. Cells that exhibited expression of less than three genes or demonstrated expression of fewer than 250 genes were excluded after a meticulous screening process. In order to quantify the proportions of rRNA and mitochondria, the methodology involved utilization of the PercentageFeatureSet function from the Seurat R package, ensuring minimization of redundancies within the analysis.

### Collection of genes associated with ferroptosis

After applying the filter for the keyword “ferroptosis” on the GENECARDS website (https://www.genecards.org/), we exclusively considered the downloaded genes that demonstrated a correlation score exceeding 1.

### scRNA-seq data processing and cell annotation

Using the “Seurat” R package, we conducted quality control on the single-cell RNA sequencing (scRNA-seq) dataset. To ensure high-quality data, we removed genes that were expressed in less than three individual cells. Additionally, cells with a gene count below 200 or above 7,000 were excluded. Furthermore, we filtered out cells with more than 10% mitochondrial genes to minimize potential technical artifacts. These rigorous filters allowed us to retain a refined and reliable scRNA-seq dataset. The remaining cells were subsequently subjected to linear regression modeling and “Log-normalization” technique for scaling and normalization to facilitate further analysis. Subsequently, the “FindVariableFeatures” tool was employed to identify the top 3,000 highly variable genes. To eliminate batch effects caused by multiple samples, the “FindIntegrationAnchors” function of the canonical correlation analysis (CCA) approach was employed. The data were consolidated and standardized using the “IntegrateData” and “ScaleData” functionalities. Principal component analysis (PCA) was then applied to reduce dimensionality and identify pivotal anchor points. Afterwards, the t-distributed stochastic neighbor embedding (t-SNE) algorithm was utilized to assess the most influential 20 principal components and identify significant clusters. To evaluate differential expression within each cluster, the “FindMarkers” tool from the “Seurat” package was employed. We employed the cutoff thresholds and adjusted criteria of *P* < 0.01 and log2 (foldchange) >0.25 to identify cluster-specific marker genes. Subsequently, the cell types were verified and annotated using the canonical marker genes of each cluster, in accordance with previous studies.

### Development and implementation of a prognostic-related model

Initially, a differential analysis was performed to identify genes that exhibited differential expression between samples with AKI and normal samples. Afterwards, the R package “survival” was utilized to conduct univariate Cox regression analysis in order to explore genes that are correlated with prognosis. These genes were later chosen for constructing a prognostic model related to cell ferroptosis, employing the Lasso and multivariate Cox regression algorithms. The effectiveness of the models was evaluated through the generation of ROC curves in order to assess their performance.

### Analysis of enrichment functional pathways

To explore the biological mechanisms and pathways associated with the two risk groups, we performed GO and KEGG enrichment analysis. Gene Set Enrichment Analysis (GSEA) was employed to investigate variations in gene function within KEGG pathways. Furthermore, we carried out additional assessment of pivotal genes. The analysis was conducted using several R packages, including “clusterProfiler,” “org.Hs.eg.db,” “enrichplot,” “dplyr,” and “ggpubr”.

### Immune cell related expression analysis

The analytical tool known as “CIBERSORT” was employed to examine the prevalence of immune cells in both the control and treatment cohorts, and the disparities in immune cell quantities between these cohorts can be visually depicted using box plots.

### Drug–gene interaction analysis

In order to explore the correlation between pharmaceuticals and genes, we leveraged the DrugBank repository to ascertain presently established or potentially linked drug compounds. Moreover, we employed the Cytoscape software to visually represent the obtained data.

### Exploration ceRNA network of the hub genes

In order to explore the cross-talk between miRNA and mRNA within the ceRNA network, we analyzed the TargetScan, miRNet, and DIANA TOOLS TarBase v.8 databases to identify relevant miRNAs targeting the central gene. To visualize the obtained information, we utilized the web-based platforms Wei Sheng Xin (http://www.bioinformatics.com.cn) and Draw Venn Diagram (http://bioinformatics.psb.ugent.be/webtools/Venn/).

### Animals and renal ischemia/reperfusion model induction

Male C57BL/6J mice aged 7 to 8 weeks were utilized for this investigation and accommodated in a 12-hour light/12-hour dark cycle. The experimental procedures were authorized by the Ethical Committee of Anhui Medical University, with certification number LLSC, 20231218. These protocols adhere to the recommendations specified in the Guide for the Care and Use of Laboratory Animals as outlined by the National Institutes of Health (NIH) in their publication no. 85–23, revised in 2011.

The induction of renal ischemia/reperfusion was conducted as follows. Initially, the mice were anesthetized by administering sodium pentobarbital at a dose of 50 mg/kg. Subsequently, the renal ischemia surgery was performed. This procedure involved a sequential incision of the skin, muscle, and fascia to expose both the left and right kidneys. Following that, the bilateral renal pedicles were clamped using noninvasive arteriole clips for a duration of 42 minutes. After the removal of the clips, reperfusion was allowed for a period of 24 hours. In comparison, the sham group underwent the same surgical procedure as the I/R group, with the exception of renal ischemia and reperfusion. After a 24-hour period, the mice were put down and kidneys were gathered. The right kidney was divided into two halves, with one being rapidly frozen for Western blot analysis, and the other being preserved in 4% phosphate-buffered formaldehyde for Immunohistochemistry (IHC). The left kidney was isolated to extract kidney cells for flow cytometry analysis.

### Western blotting

The samples were lysed using RIPA buffer supplemented with PMSF as previously described. Following that, the protein sample’s concentration was determined using the Bradford method. Subsequently, electrophoresis was performed on the samples, and they were subsequently transferred onto PVDF membranes. After blocking for 0.5 hours, the samples were subjected to incubation with ACSL4 (ab155282, Abcam, UK) at a 1:5000 dilution, SLC2A1 (21829-1-AP, Proteintech, China) at a 1:2000 dilution, The membranes were then treated with the appropriate secondary antibodies (ZB-2306/ZB-2305, ZSGB-BIO, Beijing, China) at a 1:5000 dilution for 1 hour at room temperature. Protein bands were detected using an ECL kit (Biosharp Life Sciences, BL523A, Hefei, China) and imaged with a Tanon 5200 imaging system (Tanon Technology, Shanghai, China). Densitometry analysis was conducted using the ImageJ software (ImageJ 1.4, NIH, MD, USA), utilizing β-actin as the internal reference.

### IHC in mouse kidneys

Kidneys were regularly harvested for SLC2A1 and GPX4 immunohistochemistry (IHC). Following overnight fixation in 4% paraformaldehyde, the tissues were embedded in paraffin and sectioned at a thickness of 4 μm. After subjecting the sections to antigen retrieval in a solution containing 10 mM sodium citrate, 0.05% Tween-20 at a pH of 6.0, they were heated at 95–100°C. To block endogenous peroxidase activity, the slides were treated with 3% H_2_O_2_. Additionally, 2.5% normal horse serum was applied to minimize nonspecific binding. The slides were subsequently incubated at a temperature of 4°C overnight with rabbit anti-SLC2A1 (21829-1-AP, 1:200, Proteintech), anti-GPX4 (HuaAn, Hangzhou, China) and then treated with ImmPRESS HRP polymer horse-anti-rabbit secondary antibody at room temperature for 1 hour. Negative controls were established by substituting the primary antibody with antibody diluent. The signals were visualized using the Vector^®^ DAB kit and counterstained with hematoxylin following the washing steps. To quantify the staining, a total of 20 to 30 fields (400× magnification) were randomly chosen from each section. The percentage of positive stained area was then analyzed utilizing ImageJ software.

### Determination of MDA levels of kidney tissue

MDA levels in HK-2 cell were detected using MDA activity assay kits, following the manufacturer’s protocols provided by Nanjing Jiancheng Bioengineering Institute, China (C013-2-1). MDA content was detected at a wavelength of 450 nm. Subsequently, the concentration of MDA was standardized by the total protein concentration, which was determined using the Bradford assay.

### Apoptosis and ROS measure

A flow cytometer was employed to evaluate the apoptotic rate using an Annexin V-FITC/PI Apoptosis Detection Kit (Yeasen, Shanghai, China). The proportion of cells undergoing both early and late stages of apoptosis was determined in order to calculate the overall apoptotic rate. The ROS level in HK-2 cells was measured using the ROS assay kit (Beyotime, Shanghai, China) as per the manufacturer’s instructions.

### Cell culture and drug treatment

HK2 cells were obtained from the Shanghai Institutes for Biological Sciences. The cells were cultured in DMEM-F12 (Gibco, CA, USA) supplemented with 5% FBS (Gibco, CA, USA) at a temperature of 37°C and in a 5% CO_2_-containing atmosphere. In the H/R group, the HK2 cells underwent a period of hypoxia (94% N2, 5% CO_2_, and 1% O_2_) for 24 hours, followed by reoxygenation to restore normal conditions for an additional 3 hours. The cells were then collected for further investigation.

For *in vitro* experiments, Troglitazone (4 μM, MCE) was utilized. All cells were maintained in a 37°C incubator with a 5% CO_2_ atmosphere to ensure optimal conditions.

### Transfection

The full-length SLC2A1 plasmid (obtained from Miaolingbio, Wuhan, China) was utilized for transfecting HK-2 cells, with the assistance of Polybrene A at a concentration of 5 μg/ml (Gene-Pharma, Shanghai, China), following the guidelines provided by the manufacturer.

### Transmission electron microscopy (TEM)

Tissues of renal cortical were fixed in 2.5% glutaraldehyde and then dehydrated and embedded. Firstly, 2.5% glutaraldehyde fixed for 72 h at 4°C. Then incubated at 37°C with 2% osmium tetroxide and 0.1 M cacodylate sodium (pH 7.4) for 1 h and polymerized for 48 h at 60°C. Finally, observed and evaluated with transmission electron microscopy (Hitachi, Tokyo, Japan).

### Statistical analysis

Experimental data from three independent experiments were analyzed using GraphPad Prism software (version 8.0) for biostatistics. The mean ± standard deviation (SD) was used to express the data. Group comparisons were evaluated through Student’s *t*-tests (^*^P < 0.05, ^**^P < 0.01, ^***^P < 0.001).
